# Computational analysis of flow conditions in hydrodynamic cavitation generator for water treatment processes

**DOI:** 10.1002/cjce.24572

**Published:** 2022-08-22

**Authors:** Jurij Gostiša, Primož Drešar, Marko Hočevar, Matevž Dular

**Affiliations:** ^1^ Faculty of Mechanical Engineering University of Ljubljana Ljubljana Slovenia

**Keywords:** cavitation, computational fluid dynamics, hydrodynamic cavitation generator, numerical simulation

## Abstract

The research on the potential of cavitation exploitation is currently an extremely interesting topic. To reduce the costs and time of the cavitation reactor optimization, nowadays, experimental optimization is supplemented and even replaced using computational fluid dynamics (CFD). One of the approaches towards sustainable water treatment is the use of the cavitation reactor with bluff elements mounted on its stator and rotor. The experimental results show that, besides the rotational speed, the spacing of the rotor pins has the most significant effect on the cavitation intensity and effectiveness, while the pin diameter and the surface roughness are less significant design parameters. The present paper uses a simplified CFD approach to investigate the conditions in the reactor and to select the optimal among a number of geometry variations.

## 1 INTRODUCTION

Cavitation—the appearance of vapour cavities inside an originally homogeneous liquid medium—occurs when the pressure is reduced below the vapour pressure. The liquid medium is then ‘broken’ at one or more points, and ‘cavities’ are formed whose shape depends strongly on the structure of the flow. The vapour structures are unstable, and when they reach a region of elevated pressure, they often collapse violently.^[^ ^1^ ^]^ Exploring the potential of cavitation exploitation is currently becoming an increasingly interesting topic. The availability of water is becoming an increasing problem in the globalized world, both in developed and developing countries. Therefore, an efficient and clean disinfection technology, such as the optimized use of cavitation, would be very welcome as a replacement or in combination with the existing technologies.^[^ ^2^ ^]^

Nowadays, different types of cavitation reactors are being promoted by researchers. We can generally divide them into (i) pumping and constriction reactors,^[^ ^3^, ^4^ ^]^ (ii) blow‐through reactors,^[^ ^5^, ^6^ ^]^ and (iii) rotor‐stator reactors.^[^ ^7^, ^8^, ^9^, ^10^ ^]^ Most of the advanced laboratory‐scale reactors are of the pump and constriction type, where the contaminated water (containing bacteria, viruses, algae, etc.) is forced by the pump through an orifice or venturi constriction where the sample cavitates. Blow‐through devices are essentially identical, but the sample is forced through compressed air. These devices have more controlled conditions and are suitable for scientific studies but cannot be used efficiently in industry. Finally, the most complex are the rotor‐stator devices, which are often already used in pilot tests.

One of the advantages of hydrodynamic cavitation is its scalability and potential to be used on an industrial scale. However, it is important to realize that the scaling effects and optimization are not exactly easy and inexpensive.^[^ ^11^ ^]^ Nowadays, in order to reduce the cost and time of the optimization process, experimental optimization is supplemented or even replaced by computational fluid dynamics (CFD). Simulation of the physics of cavitation dynamics, which simultaneously involves large density and compressibility fluctuations, turbulence effects, and instabilities at various scales, is still beyond the current state of the art. But in the last 20 years, engineers (mainly from the field of turbomachinery) have developed reliable methods based on CFD to predict the main features of cavitating flows.^[^ ^12^ ^]^

In the following sections, we first describe the numerical simulation of the flow in a rotating generator with hydrodynamic cavitation (RGHC). We investigate the influence of varying geometrical parameters (number and spacing of rotor pins and the gap between rotor and stator pins) on the flow characteristics inside the RGHC. From these, we select the most suitable combination and test it in the laboratory and on a pilot‐scale device for water treatment.

## 2 ROTATING GENERATOR OF HYDRODYNAMIC CAVITATION

**FIGURE 1:**
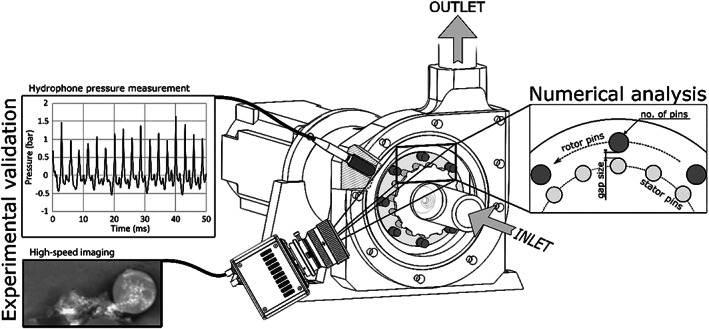
The investigated rotating generator of hydrodynamic cavitation

The investigated RGHC is a centrifugal rotor‐stator type device with bluff elements arranged on the rotor and stator perimeter (Figure 1). The fluid enters the reactor through the inlet opening at the centre of the cylindrical housing in the axial direction. After the change from the axial to the radial direction, it encounters the array of stator elements arranged on the stator disc perimeter. The stator elements are followed by the array of elements arranged on the rotating disc. After the stator and rotor cascade, the liquid exits the reactor through the outlet opening. The array of stator elements is used to reduce (dissipate) pressure in the rotor array area where hydrodynamic cavitation occurs and induces pressure fluctuations to amplify cavitation cloud collapses. Due to the centrifugal momentum introduced by the rotating array of pins, the device functions as both a cavitation generator and a pump.

This type of device was initially investigated on a pilot scale by Gostiša et al.^[^ ^9^, ^10^ ^]^ where mechanisms of cavitation generation were experimentally determined by high‐speed visualization and pressure fluctuation measurements. Principally, cavitation was found to occur behind the element, presumably in the low‐pressure wake behind, and fluctuating vapour cloud dynamics was attributed to the passing rotor‐stator interaction. Pilot‐scale experimental limitations (expensive part manufacturing) led us to make a scaled down laboratory size RGHC, where experimental analysis analogues to the pilot‐scale experiment was performed. The latter was equipped with a flow meter and pressure transducers to determine the numerical model boundary conditions and with a hydrophone to measure pressure fluctuation used to validate the numerical analysis results. Moreover, the front cover of the reactor was translucent, allowing for visualization with a high‐speed camera. The RHGC in question is schematically shown in the figure below.

The results of the studies on the pilot‐scale device^[^ ^9^, ^10^ ^]^ pointed out the number of pins as one of the most influential geometric parameters on the mechanical cavitation effects such as particle size reduction. Therefore, the number of rotor pins was selected as the first geometrical parameter. Second, gap size was also observed to have a significant effect on cavitation characteristics. It could not be directly observed in the pilot‐scale experiment but via rotor and stator pin diameter variation. Therefore, it was chosen as the second parameter for the present numerical analysis. The parameters and accompanying cases are listed in the table below.

## 3 NUMERICAL PROCEDURE

CFD software packages offer a wide range of computational models and settings that, along with the increasing computing power of an average computer, enable reliable numerical modelling of engineering problems, including the phenomenon of cavitation. Although the large‐Eddy simulation (LES) and detached‐Eddy simulation (DES) approaches are gaining importance in academia, the large computational resources required still force engineers to use a simpler Reynolds‐averaged Navier–Stokes (RANS) approach to model turbulence. Two‐phase flow is usually averaged to a homogeneous mixture flow and the mass transfer is modelled by a transport equation with source terms based on very rudimentary bubble dynamics physics.

Based on the experimental observations on a pilot‐scale device^[^ ^10^ ^]^ we focused our numerical study on the investigation of the dynamics of the interaction between the rotor and the stator (the gap between them) and the interaction among the rotor pins (the spacing between them)—the goal was to determine the details of the influences of the beforementioned parameters on the development of the cavitation cloud and the conditions in the cavitating zone. Since the focus of the interaction covers only a very small part of the device, a simplified 2D geometry was used for the simulations. This has another important consequence—since cavitation is an extremely dynamical phenomenon, time resolution is essential. Reduced computational load enabled simulations with very short time steps (0.5 μs), which further allowed the observation of shock wave dynamics inside the flow tract.

### 3.1 Governing equations

The flow is described by a set of nonlinear Navier–Stokes (NS) differential equations. Since the solution for this geometry is very hard to obtain by the direct numerical approach, a RANS modelling approach is used to tackle the closure problem. Therefore, the governing equations are proposed and written as the continuity equation:

(1) $$\frac{\partial \rho_{m}}{\partial t}+\frac{\partial }{\partial x_{i}}\left(\rho_{m}\bar{u_{i}}\right)=0,i=1,2,3$$

and momentum equation:

(2) $$\frac{\partial \left(\rho_{m}\bar{u_{i}}\right)}{\partial t}+\frac{\partial \left(\rho_{m}\bar{u_{i}}\bar{u_{j}}\right)}{\partial x_{j}}=-\frac{\partial p}{\partial x_{i}}+\frac{\partial }{\partial x_{j}}\left[\mu_{\mathrm{eff}}\left(\frac{\partial \bar{u_{i}}}{\partial x_{j}}+\frac{\partial \bar{u_{j}}}{\partial x_{i}}-\frac{2}{3}\delta_{\mathrm{ij}}\frac{\partial \bar{u_{k}}}{\partial x_{k}}\right)\right],$$

where

(3) $$\mu_{\mathrm{eff}}=\mu_{m}+\mu_{t}.$$

Note that the density and the viscosity are both defined for the mixture phase (index *m*), as explained in the section on the two‐phase flow modelling (Section 3.3). Many authors stress that for the accurate capturing of details of cavitating flow, performing a compressible flow simulation is essential.^[^ ^13^, ^14^, ^15^ ^]^ For this, one must additionally solve the energy conservation equation and introduce equations of state for vapour and liquid:

(4) $$\frac{\partial }{\partial t}\left(\rho_{m}E\right)+\frac{\partial }{\partial x_{i}}\left[u_{i}\left(\rho_{m}E+p\right)\right]=\frac{\partial }{\partial x_{j}}\left(k_{\mathrm{eff}}\frac{\partial T}{\partial x_{i}}\right),$$

where *k*_eff_ is the effective conductivity and *E* is defined as:

(5) $$E=h-\frac{p}{\rho_{m}}+\frac{1}{2}u_{i}u_{i}.$$

The variations in liquid phase density 𝜌_𝑙_ were calculated using the Tait equation as:

(6) $$\rho_{l}=\rho_{\mathrm{ref}}\sqrt[{n}]{\frac{p+B}{p_{\mathrm{ref}}+B}}$$

where 𝜌_𝑟𝑒𝑓_ and 𝑝_𝑟𝑒𝑓_ denote the reference liquid density and pressure, respectively. The values of constants 𝑛 = 7 and 𝐵 = 300 𝑀𝑃𝑎 were selected for water. The vapour fraction obeyed the ideal gas law.

### 3.2 Turbulence modelling

The two‐equation turbulent model (shear stress transport [SST] *k–ω*) is a commonly used approach in cavitation simulations. In essence, the SST *k–ω* model proposed by Menter^[^ ^16^ ^]^ blends *k–ω* by Wilcox and Chambers^[^ ^17^ ^]^ and *k–ε*, with the use of a special blending function. The function switches between the two turbulence models depending on how close to the wall of the domain the flow is. Near wall *k–ω* is enabled, on the other hand, in the outer region, *k–ε* model is enabled since it performs better away from the walls. A detailed formulation of blending functions and transport equations for extra variables of turbulent kinetic energy (*k*), turbulent frequency (*ω*) and turbulence eddy dissipation (*ε*) can be found in the literature.^[^ ^16^, ^18^, ^19^ ^]^

Specific to the SST *k–ω* model, the proper transport behaviour can be obtained by a limiter to the formulation of the eddy‐viscosity, and it is written as:

(7) $$\mu_{t}=\rho_{m}\frac{a_{1}k}{\max\left(a_{1}\omega ,SF_{2}\right)},$$

where *a*_*1*_ is a proportionality constant, *k* is turbulent kinetic energy, *ω* is turbulence frequency, *S* is an invariant measure of the strain rate tensor, and *F*_*2*_ is a function that equals 1 for boundary‐layer flows and 0 for free‐shear layers.^[^ ^16^ ^]^

### 3.3 Two‐phase flow modelling

For cavitation modelling, we use the principle of the homogeneous flow of the mixture, where the two‐phase flow is considered as a single‐phase flow of the liquid–vapour mixture. This allows us to solve only one equation of motion, as we treat the problem as single‐phase, but with variable properties of the mixture. The properties of a mixture of liquid and vapour are thus defined by the proportion of the vapour phase, using the model proposed by Bankoff.^[^ ^20^ ^]^ The density of the mixture is written:

(8) $$\rho_{m}=\alpha \rho_{v}+\left(1-\alpha \right)\rho_{l},$$

and dynamic viscosity as

(9) $$\mu_{m}=\alpha \mu_{v}+\left(1-\alpha \right)\mu_{l}.$$

In the model of the homogeneous flow of the mixture, the equations of conservation of mass and momentum are solved by the properties of the mixture, and the equation of conservation of the phase fraction must be solved:

(10) $$\frac{\partial }{\partial t}\left(\rho_{v}\alpha \right)+\nabla ∙\left(\rho_{v}\alpha u_{v}\right)=R_{e}-R_{c},$$

where *α* represents vapour volume fraction, and *R*_*e*_ and *R*_*c*_ mass transfer source terms, which account for the mass transfer between the liquid and vapour phases in cavitation and are thus connected to the growth and collapse of the vapour bubbles. Their formulation differs according to the cavitation model used.

### 3.4 Cavitation model

Mass transfer source terms are modelled based on the Rayleigh–Plesset^[^ ^21^, ^22^ ^]^ equation describing the growth of a single vapour bubble in a liquid:

(11) $$R_{b}\frac{d^{2}R_{b}}{{dt}^{2}}+\frac{3}{2}{\left(\frac{dR_{b}}{dt}\right)}^{2}=\left(\frac{p_{b}-p}{\rho_{l}}\right)-\frac{4\nu_{l}}{R_{b}}{\dot{R}}_{b}-\frac{2\sigma }{\rho_{l}R_{b}},$$

where *R*_b_ denotes bubble radius *p*_b_ bubble surface pressure, *ν*_*l*_ liquid kinematic viscosity, and *σ* liquid surface tension coefficient. In some cases, higher order terms are important,^[^ ^23^ ^]^ but, commonly, these, along with the effects of surface tension and viscosity, can be neglected. The above equation can be simplified to:

(12) $$\frac{dR_{b}}{dt}=\sqrt{\frac{2}{3}\frac{p_{b}-p}{\rho_{l}}}.$$

The above equation provides a physical approach to introduce the effects of bubble dynamics into the cavitation model. As for the Schnerr–Sauer cavitation model,^[^ ^24^ ^]^ the *R*_*e*_ and *R*_*c*_ mass transfer source terms are defined as:

when *p*_v_ ≥ *p*

(13) $$R_{e}=F_{\text{evap}}\frac{\rho_{v}\rho_{l}}{\rho }\alpha \left(1-\alpha \right)\frac{3}{R_{b}}\sqrt{\frac{2}{3}\frac{\left(p_{v}-p\right)}{\rho_{l}}},$$

when *p*_v_ ≤ *p*

(14) $$R_{c}=F_{\text{cond}}\frac{\rho_{v}\rho_{l}}{\rho }\alpha \left(1-\alpha \right)\frac{3}{R_{b}}\sqrt{\frac{2}{3}\frac{\left(p-p_{v}\right)}{\rho_{l}}},$$

where *F*_evap_ and *F*_cond_ are the empirical calibration coefficients of evaporation and condensation with the default values of the solver used 1 and 0.2, respectively.

To connect the vapour volume fraction to the number of bubbles per volume of liquid *n*_b_, the Schnerr‐Sauer cavitation model uses:

(15) $$\alpha =\frac{n_{b}\frac{4}{3}\pi R_{b}^{3}}{1+n_{b}\frac{4}{3}\pi R_{b}^{3}},$$

where bubble number density *n*_b_ is the only parameter that must be determined in this model. A default value of 10^11^ m^−3^ was used.

### 3.5 Boundary conditions

**FIGURE 2:**
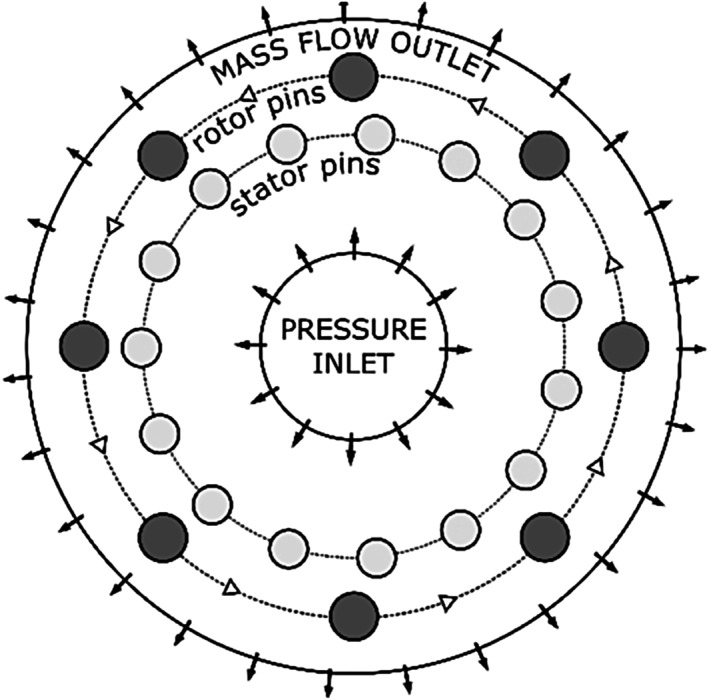
Computational domain with key components and boundary conditions

The boundary conditions of the numerical model were determined based on the measured integral hydrodynamic characteristics. Inlet pressure has a significant effect on cavitation regime/dynamics/characteristics and is not dependent on RGHC configuration but on inlet piping pressure loss. Therefore, it was fixed to −3.7 kPa gauge pressure, which is measured as the value of the inlet pressure of the non‐throttled laboratory‐scale device. The inlet vapour volume fraction was set to 0 and the turbulent intensity was set to 5%. On the contrary, the outlet pressure directly depends on the RGHC geometry due to its pumping character. That is why the mass flowrate was prescribed on the outlet boundary. The prescribed value was set to 0.4 kg/s corresponding to the flow rate of the non‐throttled laboratory‐scale device. Backflow volume fraction of the vapour phase was set to 0. Both stator and rotor pin walls were modelled with a no‐slip shear condition and a standard surface model (roughness height of 0 m and constant as 0.5). Since rotor pins are mounted on a rotating disc, a rotational velocity of 7000 rpm was prescribed with the position of the rotation axis in the centre of the disc (aligned to the centre of the computational domain). The boundary conditions are schematically shown in Figure 2.

### 3.6 Mesh

We have tested the mesh independence on five meshes. The discretization errors of 4% and 0.7% were determined by Richardson extrapolation^[^ ^25^ ^]^ against the average pressure difference Δ*p* (Table 1) for the mid‐course mesh and fine mesh, respectively.

**TABLE 1:** Mesh independence study

Number of mesh cells	Δ*p* (bar)
90 000	0.92
140 000	1.02
235 000	1.15
330 000	1.19
500 000	1.20

**FIGURE 3:**
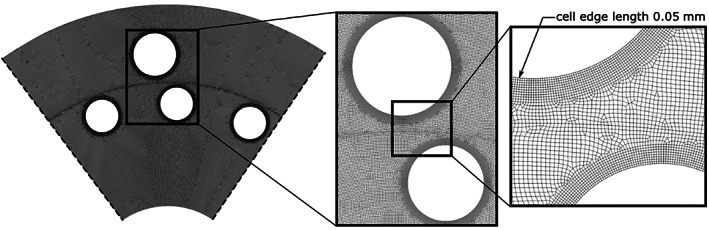
Global (left) and a detailed view of the mesh in the rotor‐stator gap (right)

The mesh we chose for the analysis consists of 330 000 cells with cell width set to 0.05 mm at the edge of the pins (detail in Figure 3). That results in approximately 30 cells across the gap when the rotor and stator pin are aligned. In the region of highest pressure and velocity gradients (in the immediate proximity of the pins) a refined mesh of 10 layered cells with a total thickens 0.5 mm was used.

### 3.7 Physics and solver settings

Numerical simulations were performed using time‐dependent Reynolds‐averaged Navier–Stokes equations implemented in Ansys Fluent 2021R1 solver. A homogeneous mixture of water and water vapour was considered, and a Schnerr‐Sauer cavitation model^[^ ^24^ ^]^ with an evaporation pressure of 3540 Pa and the bubble number density of 10^11^ m^−3^ was used. For the turbulent model, a modified SST *k–ω* model was used. The pressure‐implicit with splitting of operators (PISO) algorithm^[^ ^26^ ^]^ was used to couple the pressure and velocity. Generally, we used the second‐order upwind scheme,^[^ ^27^ ^]^ for spatial discretization of partial differential equations, which gives more accurate results with slightly higher consumption of computer resources compared to the first‐order upwind scheme. Only in the case of the volume fraction equation, the first‐order upwind scheme was used to achieve better stability. The PREssure STaggering Option (PRESTO)! interpolation scheme was used to discretize the pressure,^[^ ^28^ ^]^ and for the discretization of the volume fraction first‐order upwind scheme^[^ ^27^ ^]^ was used. For transient solutions, time integration was calculated using the Bounded second‐order implicit transient formulation.

The convergence criterion was determined by observing the evolution of various flow parameters, such as inlet and outlet velocities, absolute pressure, and mass‐flow balance in the computational domain. The monitored flow parameters were always converged after the sum of the imbalance of the transport equations between iterations over all cells in the computational domain fell below 10^−4^ of the iterative numerical solution of the individual equations in each time step of the simulation. The iteration error of less than 0.2% was estimated. The size of the time step of 0.5 μs was used, and results were saved with a 1 μs increment to allow for the shockwave propagation evaluation. After initial calculation for the period of multiple rotor rotations, a period of a single rotation (duration of 8 ms) was found to be sufficient for the cavitation cloud dynamics description.

## 4 RESULTS

We show and discuss the results of simulations for the five cases, highlighted in Table 2. We first discuss the specifics of cavitation dynamics and shock waves. Both the appearance of large vapour structures and the rapid passing of the high‐pressure wave were reported as possible causes for contaminant destruction in the literature.^[^ ^4^, ^29^, ^30^ ^]^ Then, a closer look at the local shear is provided, again due to the recent reports on it possibly being a needed condition for contaminant destruction. Finally, a short discussion on the guidelines for further optimization of the rotor‐stator geometry is given. We consider the first case—rotor with 8 pins and a 1.75 mm gap between rotor and stator pins as the representative geometry against which the other geometries are compared.

**TABLE 2:** Variation of the influential geometrical parameters

Case no,	Case id	No. pins	Gap size (mm)
1	8 pin	8	1.75
2	12 pin	12	1.75
3	16 pin	16	1.75
4	4.25 gap	8	4.25
5	0.25 gap	8	0.25

### 4.1 Cavitation dynamics

First, we take a closer look at the vapour cloud dynamics during the rotor‐stator pin pass. The cavitating conditions can already be roughly evaluated based on integral vapour area across the entire domain over the entire calculation time as listed in the Table 3. The results show significantly higher vapour fractions in the cases with 8 pin, 12 pin, and 0.25 gap, than in the cases of 16 pin and 4.25 gap. Meaning, that significantly more vapour (hence cavitation events) is present in the latter cases. This can be also seen later in Figure 5, where the evolution of the vaporous clouds is observed for each case.

**TABLE 3:** Vapour fraction average across entire area and over total calculation time

Case id	Average vapour area (mm^2^)
8 pin	106.4
12 pin	108.1
16 pin	40.3
4.25 gap	22.0
0.25 gap	206.1

**FIGURE 4:**
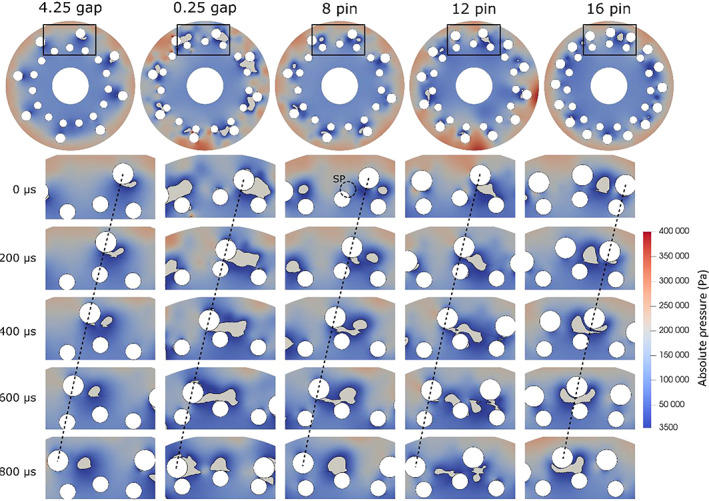
Cavitation behaviour, with corresponding pressure field, during the passing of the rotor stator pins for the five cases. SP, stagnation point

To get a deeper insight into cavitation generation mechanisms, a part of the domain was isolated, and the vapour cloud dynamics was investigated (Figure 4). For each case, 800 μs of flow time is shown. The grey area represents 10% isosurface of the vapour volume fraction based on reports of experimental measurements^[^ ^31^, ^32^ ^]^ and the fact that this is the most commonly used value in the studies (also, somewhat different threshold values do not drastically change the cavitation cloud appearance). Also shown is the local pressure field. The rotor pins rotate in the counter‐clockwise direction.

A general (common for all five cases) observation of a single, representative, rotor‐stator pin pass (Figure 4), we can see that the vapour cloud is continuously present behind the rotor pin, independently of the relative position to the stator pin. Furthermore, one can see that as the rotor pin is approaching the stator pin, the gap between the two is shrinking and thus pressure is slowly increasing (best seen for the case with 8 pins). Due to the streamline pattern, a stagnation point (SP) occurs on the stator pin side, facing the approaching rotor pin (for clarity, this region is indicated by ‘SP’ in the top image for the 8 pin case). When they meet (when the rotor pin passes the SP of the stator pin), the pressure distribution suddenly changes, and in the gap between the pins, a low‐pressure area forms and cavitates. Later, when the rotor pin is moving away from the stator pin, the gap between the two is expanding and thus the pressure in that area is decreasing. While the low‐pressure area (the wake behind the stator pin) is stretching along the path of the pin rotation and the vapour cloud is stretched along it. During this period, the area of the vapour cloud increases. Finally, the rotor pin moves away to the point that the interaction is lost, the cloud ceases to grow and begins to collapse. In some cases, a secondary low‐pressure area behind the pin can form, hence the vapour cloud detaches from the rotor pin wall and settles inside the secondary low‐pressure area.

In the case of an enlarged gap between the rotor and the stator (case of 4.25 gap), cloud stretching in the wake of the rotor pin was not observed. This is due to the less pronounced streamline pattern and thus the less abrupt pressure change at the SP passing, as described above. Similarly, the case of 16 pins also exhibits less pronounced cloud stretching and, in some instances, even cloud throttling, which is attributed to the short spacing between the rotor pins. Because of that, a sufficiently low‐pressure wake is not able to form and hence the cavitation cloud growth is restricted. At a smaller size, the cavitation dynamics in the growth phase, is still comparable to the 8 and 12 pin cases, while the stretching and detaching of the cloud are less likely to occur. Finally, we can observe that increasing the number of pins from 8 to 12 does not change the topology or the dynamics of cavitation significantly. On the other hand, decreasing the gap from 1.75 mm to only 0.25 mm causes more abrupt pressure change at the passing of the rotor and consequently distinctly prolonged vapour clouds.

**FIGURE 5:**
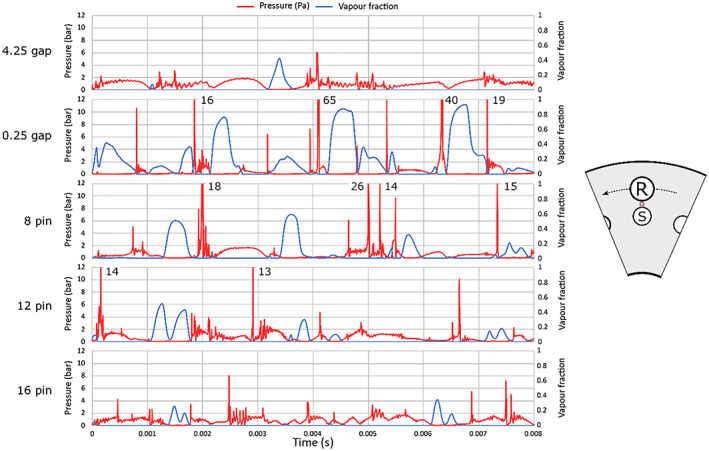
Time evolution of vapour volume fraction and accompanying average absolute pressure in the marked area for the five cases

Further on, temporal pressure and volume fraction dynamics was investigated in the gap area between the rotor and stator. The diagrams in Figure 5 show time series of the average absolute pressure and vapour fraction in the area marked with red circle (left in Figure 5). Note that some values of peak pressure are clipped, and peak values are marked to allow for better clarity (8 and 12 pin rotor and the case with the smallest gap—0.25 mm).

We can notice that the pressure peaks at the moment when the vapour cloud collapses (when the vapour volume fraction drops to zero), which indicates the emission of the shockwave at this moment. Sometimes, a delay occurs between the point where the vapour fraction reaches zero and the pressure peak. The delay is attributed to the collapse happening downstream of the observed point, such as in the case of 8 pin at 0.0015 s. In this instance, the cloud has not yet fully collapsed when the observed vapour reaches zero as the centre of the collapse is not aligned with the observed point. Since the pressure peak occurs at the collapse, the delay between the vapour fraction zero and the pressure peak can be observed. The cloud sometimes even sticks to the stator pin and collapses after being extensively prolonged. In this case, an even longer delay can be observed, as in the case of 8 pin at a time of 0.004 s.

The pressure peaks, indicating shockwave emissions, are most pronounced in the case of 8 and 12 pin rotor and even more in the case of the smallest rotor stator gap (0.25 mm). Not only the number but also the peak pressure values are higher in these three cases. Interestingly, regarding the number of pressure peaks, the case with 8 pins seems to be favourable over the case of 12 pins. As previously shown in Table 3, where the amount of the vapour present in the complete domain was discussed, the amount of vapour in the smaller domain shows the same trend—ascending area order: 4.25 mm gap, 16 pin, similar 8 and 12 pin, and 0.25 mm gap.

### 4.2 Time evolution of pressure waves

**FIGURE 6:**
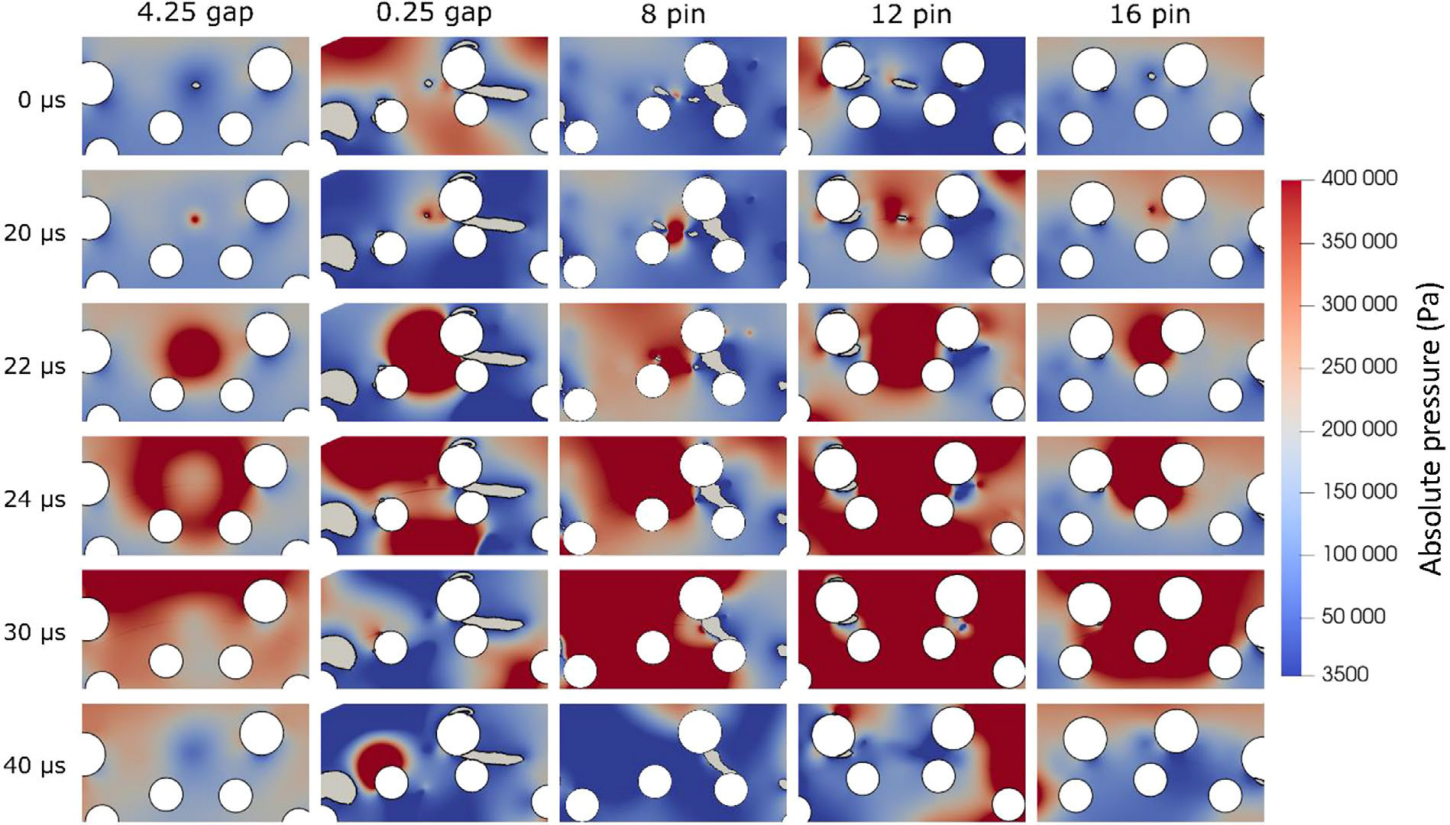
Pressure dynamics after the collapse of the cavitation cloud are shown for the five cases

Since liquid and vapour phases were both modelled as compressible fluids, pressure wave formation and propagation could be investigated. As previously mentioned, pressure waves were attributed to cloud collapse and were indicated by discrete pressure peaks in the time series of pressure averaged in the small area between the rotor and stator pins. Further on, the pressure wave is visually evaluated as shown in Figure 6. A time series, of images starting with the vapour cloud at its latest stage of collapse, followed by shock initialization at the collapse, pressure wave propagation, and dissipation at the end, is shown. The whole sequence is 40 μs long. Please note that the time difference between consecutive images is not constant. This was done for the sake of clarity, as the collapse is a much longer process than shock wave propagation.

One can see that a shockwave forms when the vapour cloud collapses and propagates with the sonic velocity (roughly 1500 m/s) across the domain. Two types of collapse can be distinguished, that is, the collapse of spherical and elongated clouds. Figure 6 shows spherical cloud collapse for 4.25 mm gap, 0.25 mm gap, and 16 pin cases and elongated cloud collapse in the cases of 8 and 12 pins.

An interesting observation can be seen in the case of a 0.25 mm gap, where a spherical cavity collapse triggered a shockwave that collapsed a secondary cavity (positioned relatively far away from the primary one—on the stator pin wall), which again emitted a shock wave.

**FIGURE 7:**
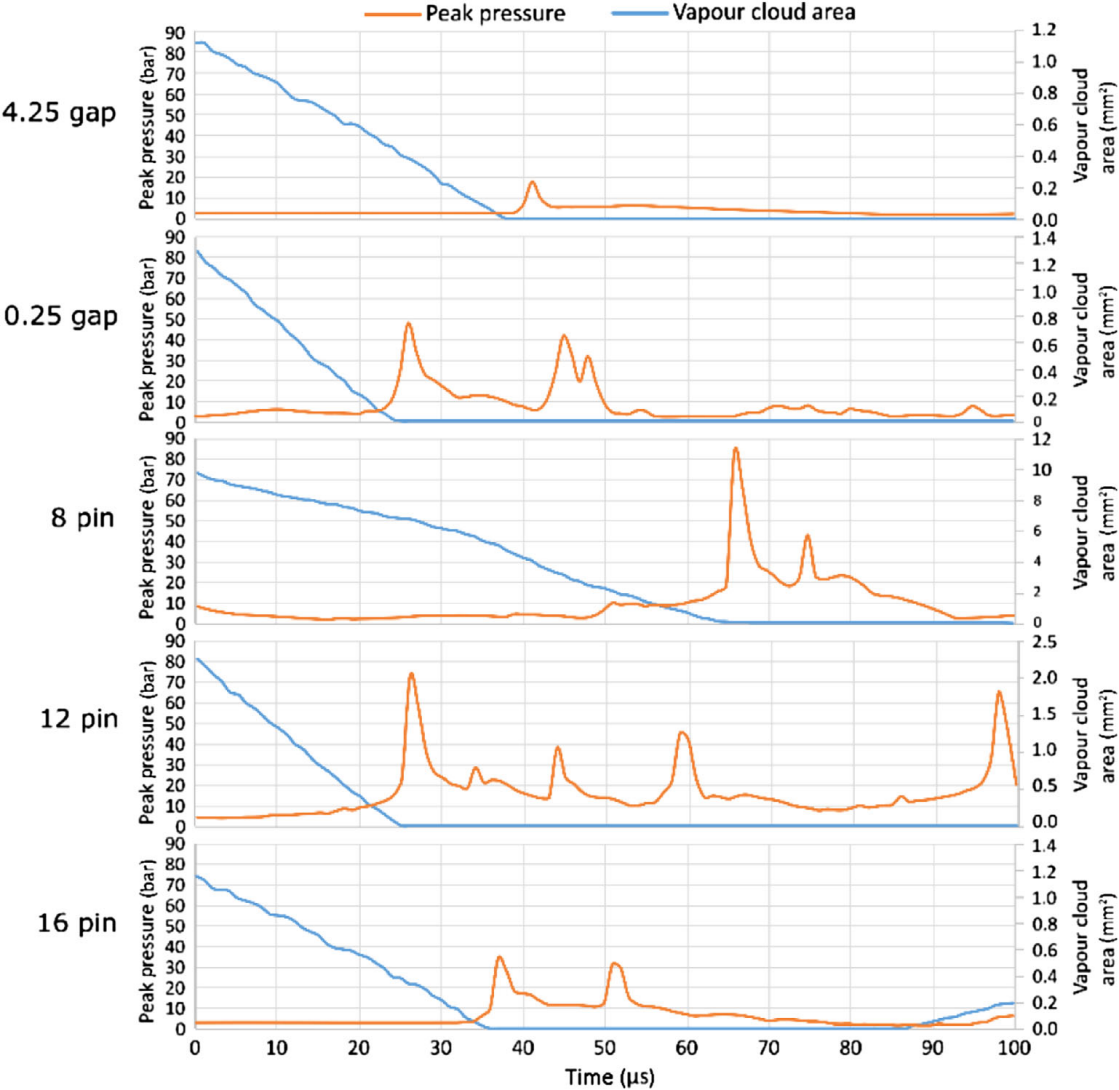
Time evolution of pressure and the corresponding vapour volume fraction in the marked area for the five cases

Since a field image cannot cover the whole amplitude of the pressure peak, an average pressure in a small area is plotted in a diagram form and shown in Figure 7.

Looking at both Figures 6 and 7, we see that higher peak pressure values are observed in the case of elongated cloud collapses (cases with 8 and 12 pins). This is a similar result as obtained from the analysis, where also the case with a 0.25 mm gap showed high peak pressures. Nonetheless, we can expect that the origin of the high amplitude shock waves is predominantly the collapse of an elongated cavity.

### 4.3 Time evolution of shear

Recent experimental and numerical studies on the interaction between bubbles on one side and a solid object, liposome, or bacterium on the other side point to the high shear as the dominant mechanism of contaminant destruction.^[^ ^30^, ^33^, ^34^, ^35^, ^36^ ^]^ The shear is most simply shown by the strain rate magnitude field. The strain rate tensor definition is written as:

$$S_{\mathrm{ij}}=\frac{1}{2}\left(\frac{\partial u_{i}}{\partial x_{j}}+\frac{\partial u_{j}}{\partial x_{i}}-\frac{2}{3}\delta_{\mathrm{ij}}\frac{\partial \bar{u_{k}}}{\partial x_{k}}\right).$$

where strain rate magnitude is defined as:

$$S=\sqrt{2S_{\mathrm{ij}}S_{\mathrm{ij}}}.$$

**FIGURE 8:**
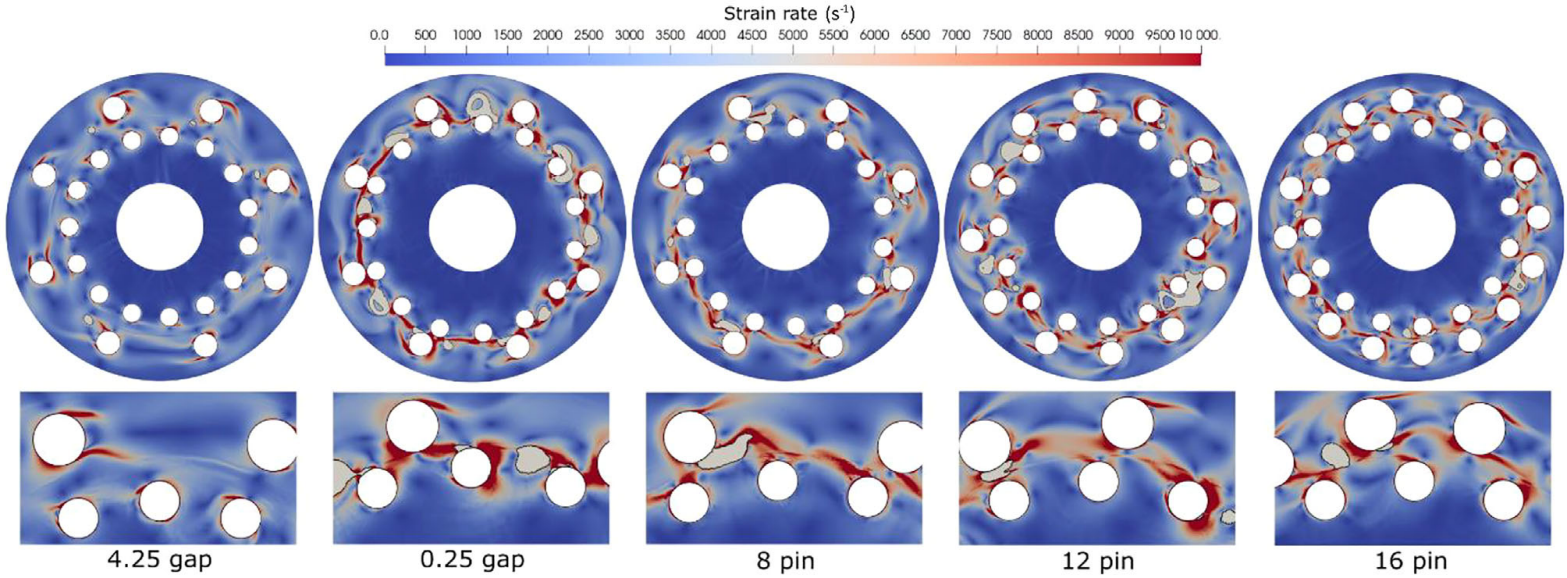
Strain rate magnitude distribution dynamics during the passing of the rotor stator pins for the five cases

Figure 8 shows the strain rate magnitude distribution across the entire computational domain and, in detail, in the rotor‐stator pin interaction area. The temporal evolution of the strain rate magnitude field is not significant, hence only one time instance is shown (refer to Video S1).

**FIGURE 9:**
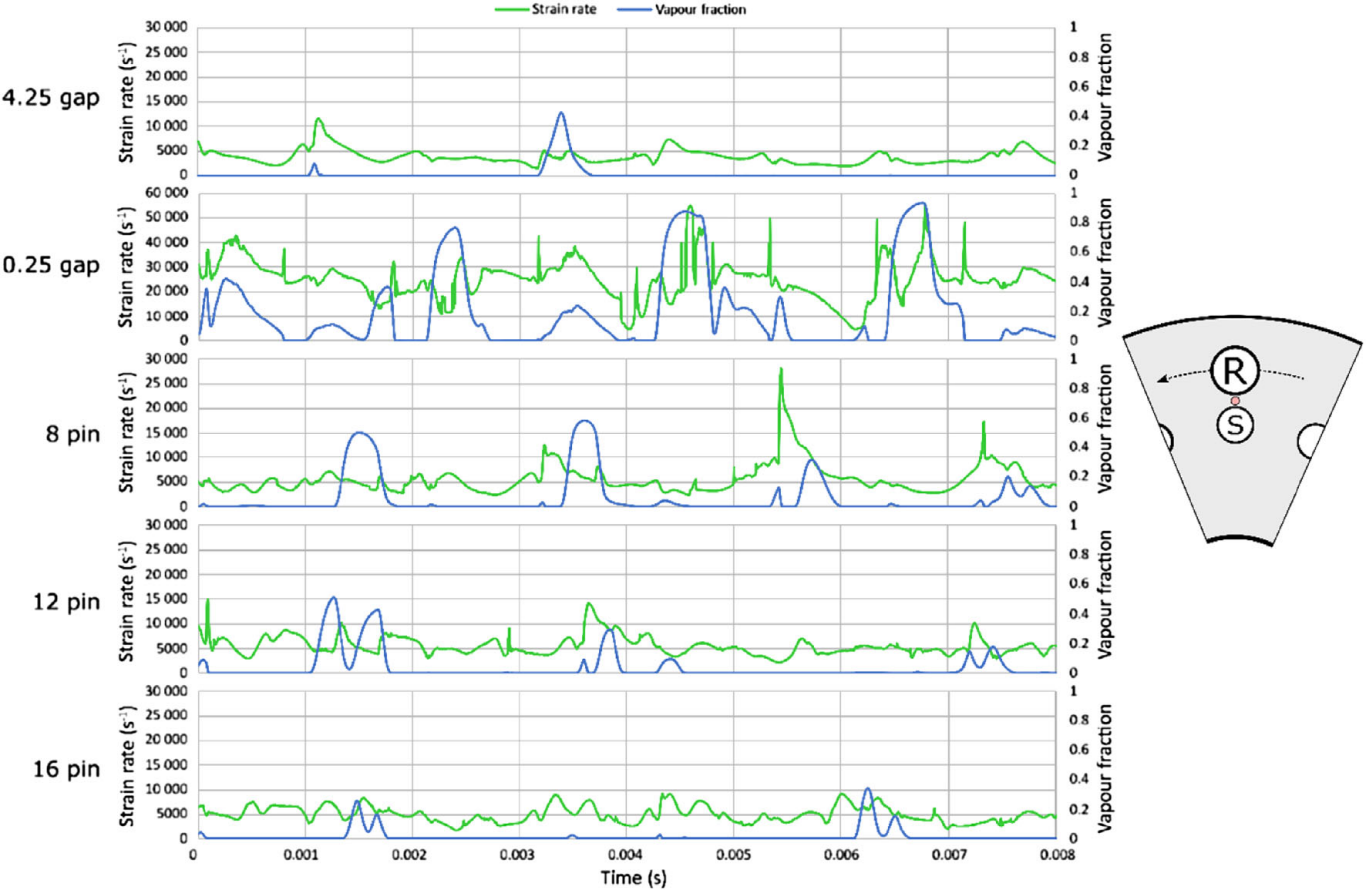
Time evolution of shear and the corresponding vapour volume fraction in the marked area for the five cases

The strain rate field exhibits areas of increased strain magnitude behind both stator and rotor pin edges, on the ‘edge’ of the low‐pressure wake behind the pin. The ‘hair‐like’ shaped area of high strain is oriented in the direction opposite to the pin movement. Typical for all cases, except the 4.25 gap is higher strain between the rotor and stator pins, affected by the interaction of the two. Regarding the number of the pins, the hair‐like area of increased strain occurs on every rotor and stator pin, and based on that, we could conclude that the area of the increased strain is higher in the cases of 12 and 16 pin compared to 8 pin. As expected, one observes higher strain rate magnitude in the case of the smallest gap between the rotor and the stator discs (0.25 gap case). Still, the other cases, apart from the one with the largest gap, show the strain to be in the same order of magnitude. Interesting is also a closer observation of the strain rate evolution in a small area between the rotor and the stator discs, and the influence of cavitation itself on its evolution (Figure 9).

A correlation between the local vapour volume fraction and the strain rate magnitude can be seen. This is most apparent for the cases of the smallest gap (0.25 gap case) and the 8 pin rotor case. We see that the strain rate peaks as cavitation appears (denoted by the high vapour volume fraction). This particular result shows that cavitation triggers high strain. And it indirectly confirms our previous experimental and numerical observations that cavitation‐triggered high strain rate causes the destruction of contaminants.^[^ ^30^, ^33^, ^34^, ^35^, ^36^ ^]^

Based on the results interpretation, the optimization should proceed in the direction of shortening the rotor‐stator gap and not increasing the rotor pin number or shortening the distance between to consequent rotor elements. The cases with 8/12 pins and the shortest rotor‐stator gap exhibit more cavitation events, namely, vapour cloud onset, growth, and collapse, indicated by larger overall vapour fraction in the cases. Moreover, cavitation cloud collapses are shown to cause high pressure pulses, indicated as discrete pressure peaks in the graph in Figure 5. Thus, highest peak amplitudes were identified in the cases of 0.25 gap and 8 pin (Figure 7). Some high amplitude peaks were identified also in the case of 12 pin, hence the latter should be considered for further investigation. Elongated (non‐spherical) clouds were found to induce pressure shockwaves with higher amplitudes and such clouds were found in the cases of 8 and 12 pin. Comparatively higher strain rate values, indicating higher shear stress fields, were indicated in the case with the tightest rotor‐stator gap. Hence, gap shortening should be considered in further development. While the investigated RGHC is a laboratory‐scale size device, optimal pin number should be a part of scale‐up investigation. The premise is to achieve a sufficiently large low‐pressure area behind the rotor bluff element and avoid throttling with the consequent element.

## 5 EXPERIMENTAL VALIDATION

Experimentally obtained data was first used to determine the calculation boundary conditions, and second, validation was performed based on the pressure fluctuation measurement and cavitation phenomena visualization.

### 5.1 Brief description of the experiment

**FIGURE 10:**
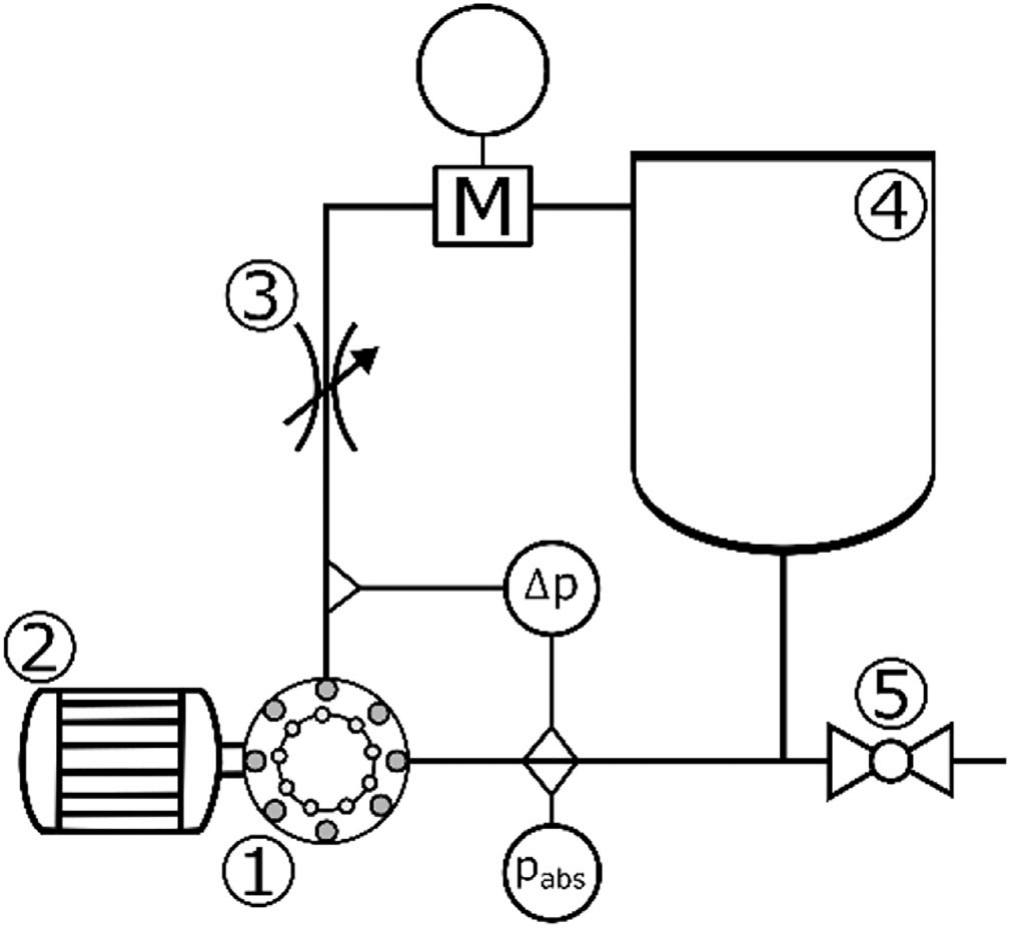
Laboratory‐scale test rig with key components: 1–RGHC, 2–servo motor, 3–throttle valve, 4–reservoir, 5–discharge valve, pabs–absolute pressure transducer, Δp–differential pressure transducer, and M–electromagnetic flow metre

The experiment was carried out on a laboratory‐scale test rig, where all key integral hydrodynamic characteristics can be measured while allowing for simultaneous visualization and pressure fluctuation measurement. The test rig is schematically shown in Figure 10 and consists of the RGHC (Figure 1, 10), driven by a 1.5 kW servo motor (Figure 2, 10) capable of setting precise rotation speeds of up to 8000 rpm and torque measurement. The RGHC is installed in a closed‐circuit pipeline with a throttle valve (Figure 3, 10) on the high‐pressure side used to set the pressure load of the system and a 5 L reservoir (Figure 4, 10). Samples of the processed media can be taken via the discharge valve (Figure 5, 10). The pressure difference across the RGHC is measured with a differential pressure transducer (Figure 10‐Δ*p*), the inlet pressure with an absolute pressure transducer (Figure 10‐*p*_abs_) and the flow rate with an electromagnetic flow metre (Figure 10‐M).

The RGHC is equipped with a translucent cover, allowing for the visualization of the cavitation phenomena in the rotor‐stator area. The latter was carried out with the Photron Fishcam SA‐Z high‐speed camera, utilizing a framerate of 150 000 fps at a resolution of 384 × 265 px. High frame rate and wide field of view were chosen at the cost of lower resolution to allow for high temporal resolution and longer pin path track. Simultaneously, pressure fluctuation measurement was performed using the Teledyne Reason TC4013 hydrophone with a sampling frequency of 150 kHz. The camera and hydrophone were installed as shown in Figure 1.

### 5.2 Results

**FIGURE 11:**
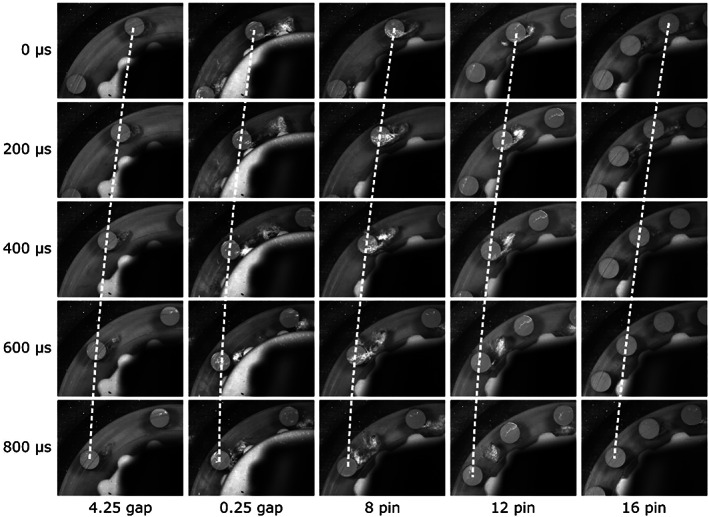
Cavitation behaviour during the passing of the rotor stator pins for the five cases

Based on the qualitative cavitation cloud dynamics comparison, similarities to the calculated results can be observed. Cavitation cloud dynamics was experimentally evaluated as shown in the work by Gostiša et al.^[^ ^10^ ^]^ The image was first rotated around the disc centre of rotation, and the cloud area was extracted from the image with an algorithm that includes mean background subtraction, pin masking, greyscale thresholding, and morphological closing. The overall trend of vapour fraction was found to be higher in the cases of 0.25 gap, 8 pin, and 12 pin compared to lower vapour fractions in the other two cases. Furthermore, the cavitation cloud onset in the gap at the time when the rotor pin passes the stator one (Figure 11 at 400 μs) is very distinct. The gap seems to have a negligible effect on the vapour cloud formation in the case of 4.25 gap, compared to a much more significant effect in the case of 0.25 gap. The 8 and 12 pin cases exhibit distinct cloud prolongation when the rotor pin moves away from the stator pin (Figure 11 at 600 μs), corresponding to the mechanism of an enlarging low‐pressure area with a distancing rotor pin.

**FIGURE 12:**
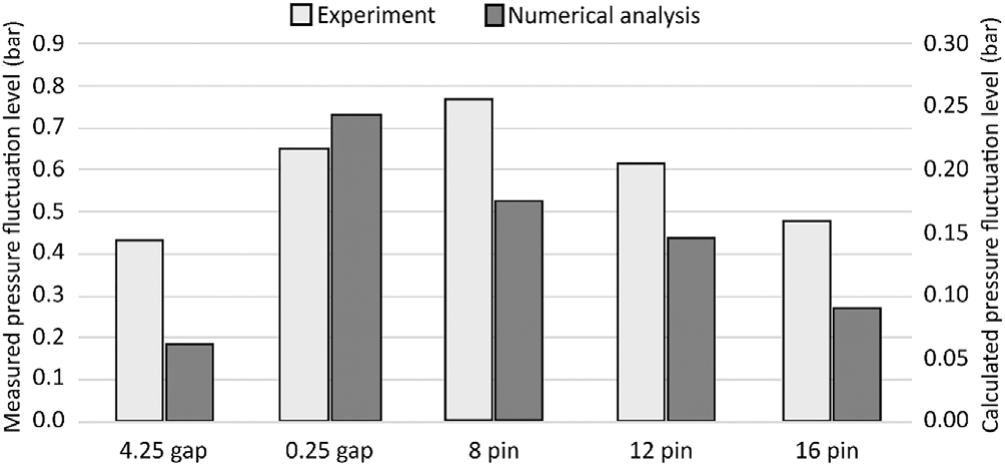
Calculated and measured pressure fluctuation levels comparison

Figure 12 depicts the calculated and measured pressure fluctuation levels. The measured levels are approximately four times higher than the calculated ones. This discrepancy can mainly be attributed mainly to the effects of hydrophone mounting and housing vibrations originating from the motor and power transmission, but also to the averaging of the pressure magnitude in the simulation. One of the influential contributions to the deviation from the experimentally obtained data could be the 2D simplification of the model. Although the geometry of the RGHC is predominantly planar, the cavitation cloud is not. Moreover, the scale difference between the cavitation cloud and the cavitation bubbles, which form the cloud, impedes us from describing the phenomena inside the cloud.

The trends of numerically obtained and measured pressure fluctuation levels are very similar. Decreasing pressure fluctuation levels with increasing pin number and increasing levels with decreasing pin gap size can be observed. The only exception is the case of 0.25 gap, where the numerically obtained pressure fluctuation is relatively higher than the measured one. Interestingly, the fluctuation level measured in the case of 8 pin (gap size 1.75 mm) is higher than the one measured in the case of 0.25 gap. The latter could indicate the significance of not only pin proximity but also cavitation cloud collapse dynamics in the pressure fluctuation.

## 6 CONCLUSIONS AND FURTHER WORK

A simplified numerical model of the RGHC has been developed and verified with experimentally obtained data. Vapour fraction, pressure, and strain rate dynamics were used to quantify the cavitation intensity and shockwave magnitudes were related to vapour cloud collapse types. Geometrical characteristics number of rotor pins and rotor‐stator gap size were investigated, where lower pin number and smaller gap size were found to have favourable effects on cavitation properties.

A future study investigating the link between cavitation properties and effects on the contaminated samples, such as particle size reduction, chemical properties, settleability, etc., is planned. The newly developed tool will be used to evaluate further RGHC improvements in the direction of increased pressure fluctuation level and the number of cavitation events.

**FIGURE 13:**
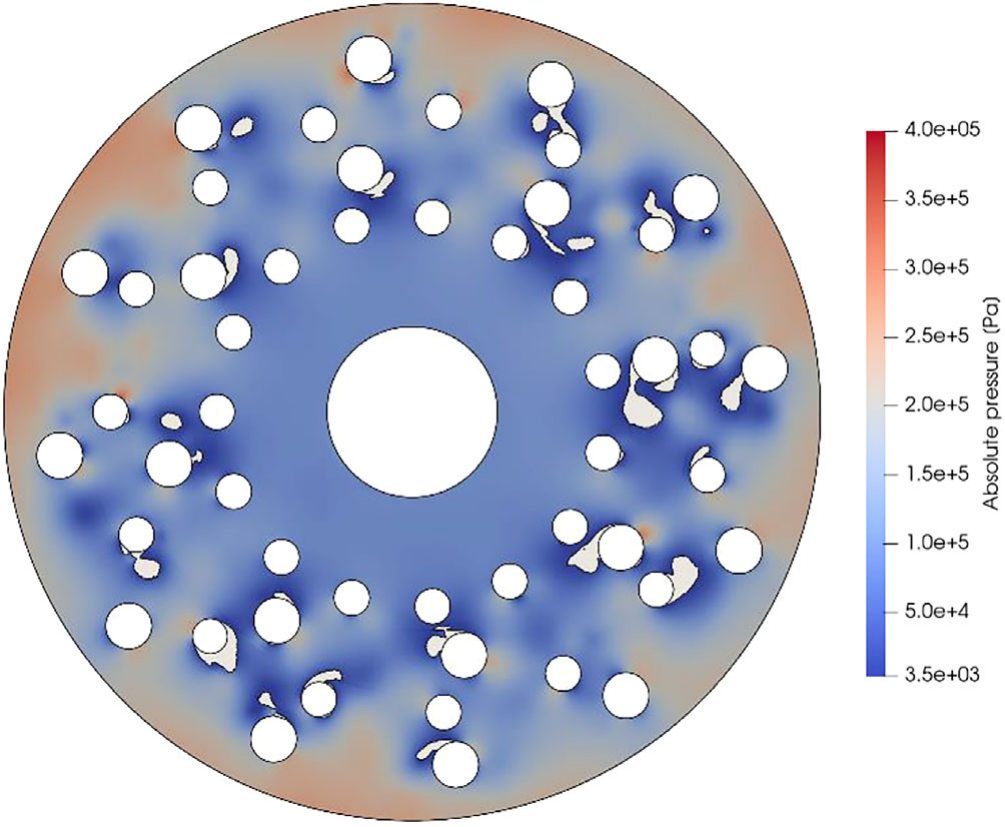
Preliminary results of the multiple rotor‐stator cascade geometry rotating generator with hydrodynamic cavitation (RGHC)

One of the immediate next steps will be the stator pin number and rotor pin shape effect investigation, while triangular shapes returned promising experimental results. Solely, shape variation might be promising, but it is not likely to increase the number of cavitation events for a factor. That could be achieved by a multiple rotor‐stator cascade device type (Figure 13). Preliminary calculations have already been made with the modelling procedure described in this study, proving it suitable for evaluation of such geometry types.

## AUTHOR CONTRIBUTIONS

**Jurij Gostiša:** Conceptualization; investigation; methodology; visualization; writing – original draft; writing – review and editing. **Primož Drešar:** Methodology; writing – original draft. **Marko Hočevar:** Conceptualization; methodology; supervision; writing – original draft. **Matevž Dular:** Conceptualization; funding acquisition; investigation; methodology; supervision; writing – original draft; writing – review and editing.

## CONFLICT OF INTEREST

The authors declare that they have no known competing financial interests or personal relationships that could have appeared to influence the work reported in this paper.

### PEER REVIEW

The peer review history for this article is available at https://publons.com/publon/10.1002/cjce.24572.

## Supporting information


**Video S1** Supplementary Videos

## Data Availability

The data that support the findings of this study are available from the corresponding author upon reasonable request.
